# Mitosis in circulating tumor cells stratifies highly aggressive breast carcinomas

**DOI:** 10.1186/s13058-016-0706-4

**Published:** 2016-05-04

**Authors:** Daniel L. Adams, Diane K. Adams, Steingrimur Stefansson, Christian Haudenschild, Stuart S. Martin, Monica Charpentier, Saranya Chumsri, Massimo Cristofanilli, Cha-Mei Tang, R. Katherine Alpaugh

**Affiliations:** Creatv MicroTech, Inc., 11 Deer Park Dr., Monmouth Junction, NJ 08852 USA; Rutgers, the State University of New Jersey, 71 Dudley Rd, New Brunswick, NJ 08901 USA; HeMemics Biotechnologies Inc., 9700 Great Seneca Highway, Rockville, MD 20850 USA; George Washington University Medical Center, 2121 Eye Street, NW, Washington, DC 20052 USA; University of Maryland Baltimore Greenebaum Cancer Center, 655 W. Baltimore St., Baltimore, MD 21136 USA; Mayo Clinic Cancer Center, 4500 San Pablo Rd., Jacksonville, FL 32224 USA; Robert H Lurie Comprehensive Cancer Center, Northwestern University, 645 N Michigan Avenue, Chicago, IL 60611 USA; Creatv MicroTech, Inc., 11609 Lake Potomac Drive, Potomac, MD 20854 USA; Fox Chase Cancer Center, Protocol Support Laboratory, 333 Cottman Ave., Philadelphia, PA 19111 USA

**Keywords:** Circulating tumor cells, Mitotic index of CTCs, Blood based biopsy, Breast cancer cell motility

## Abstract

**Background:**

Enumeration of circulating tumor cells (CTCs) isolated from the peripheral blood of breast cancer patients holds promise as a clinically relevant, minimally invasive diagnostic test. However, CTC utility has been limited as a prognostic indicator of survival by the inability to stratify patients beyond general enumeration. In comparison, histological biopsy examinations remain the standard method for confirming malignancy and grading malignant cells, allowing for cancer identification and then assessing patient cohorts for prognostic and predictive value. Typically, CTC identification relies on immunofluorescent staining assessed as absent/present, which is somewhat subjective and limited in its ability to characterize these cells. In contrast, the physical features used in histological cytology comprise the gold standard method used to identify and preliminarily characterize the cancer cells. Here, we superimpose the methods, cytologically subtyping CTCs labeled with immunohistochemical fluorescence stains to improve their prognostic value in relation to survival.

**Methods:**

In this single-blind prospective pilot study, we tracked 36 patients with late-stage breast cancer over 24 months to compare overall survival between simple CTC enumeration and subtyping mitotic CTCs. A power analysis (1-β = 0. 9, α = 0.05) determined that a pilot size of 30 patients was sufficient to stratify this patient cohort; 36 in total were enrolled.

**Results:**

Our results confirmed that CTC number is a prognostic indicator of patient survival, with a hazard ratio 5.2, *p* = 0.005 (95 % CI 1.6–16.5). However, by simply subtyping the same population based on CTCs in cytological mitosis, the hazard ratio increased dramatically to 11.1, *p* < 0.001 (95 % CI 3.1–39.7).

**Conclusions:**

Our data suggest that (1) mitotic CTCs are relativity common in aggressive late-stage breast cancer, (2) mitotic CTCs may significantly correlate with shortened overall survival, and (3) larger and more defined patient cohort studies are clearly called for based on this initial pilot study.

**Electronic supplementary material:**

The online version of this article (doi:10.1186/s13058-016-0706-4) contains supplementary material, which is available to authorized users.

## Background

Using circulating tumor cells (CTCs) in patient stratification has served as a noninvasive blood biomarker for prognosis and response to therapy in late-stage cancer, though the actual utility of CTCs remains largely academic [1, 2]. A primary reason for limited clinical use of CTCs is the inability to translate prognostic applications into mainstream clinical treatment [1, 2]. This is due to a variety of reasons, including CTC rarity and the current inability to accurately distinguish highly aggressive from less aggressive cells [2–4]. In comparison, pathological grading for cell differentiation, including assessing the mitotic index (MI) in tumor tissue biopsies, is the gold standard in cancer diagnosis, prognosis, and treatment and is an intricate part of tumor-staging algorithms [1, 5–9]. We hypothesized that, like tissue biopsies, the inclusion of a mitotic cell count along with the standard CTC enumeration may identify more aggressive CTC populations and provide a more accurate prognostic stratification of patients with breast cancer.

Cancer is graded by histopathological examination of a tissue biopsy extracted from suspicious tissue samples, primarily for cancer diagnosis [1, 5–9]. Histopathological examination also allows for stratification of patients based on the morphology of the cells from the tissue (i.e., cell grade) for aiding patient assessment and treatment [1, 5–8]. Though many cancers are graded using differing grading systems (e.g., Gleason, Bloom and Richardson Nottingham) and there are issues with subjectivity and tumor heterogeneity, certain aspects are universal in the pathological assessment of malignancy (i.e., MI and cell differentiation) [1, 5–9]. Mitosis in tumor cell grading, identified by specific cellular events occurring during cell division (e.g., prophase, metaphase, anaphase, telophase [5, 9, 10]), is considered a primary predictor of survival and an indicator of therapy response [5–10].

In CTCs, the inability to provide detailed cytological assessment in cells has led some groups to rely on the related [11, 12], yet biologically independent [13, 14], proliferation index (PI) in their quantification (i.e., methylation-inhibited binding protein 1 (clone MIB-1), proliferating cell nuclear antigen (PCNA), Ki-67, etc.). However, the use of PI biomarkers in cancer is highly contested and controversial, and the quantification and prognostic value of these markers is different to those of mitosis [13, 14]. Given that there has been at least one case study describing the visualization of mitosis in CTCs [15], we wished to examine whether the more basic visual cytological assessment of cells in mitosis is applicable to CTCs and whether this information gives similar clinical information to standard pathological assessment [11–16].

CTCs are found in approximately 65–85 % of patients with metastatic disease, and CTC enumeration is an independent prognostic indicator of survival (i.e., high number of CTCs equates to shorter survival) [2–4, 17–20]. As cancer grading is a form of morphological classification, it has been suggested that characterizing additional CTC phenotypes may provide additional prognostic stratification in patients with cancer [3, 21–23]. Recently, we have shown that filter-based isolation of CTCs from peripheral blood retains detailed cellular architecture, allowing for a more descriptive assessment of CTCs (i.e., apoptotic CTCs and dividing CTCs) [3, 17, 18, 24]. Classification of filtered tumor cells is currently used by histopathologists to identify and grade cancer cells from a number of body fluids including urine (bladder) [10], lung aspirates (lung) [25], and cerebral spinal fluid (brain/neuron) [26], though it is not commonly used for blood-based biopsies [3].

In this prospective pilot study, we examined the morphology of CTCs in the peripheral blood of patients with metastatic breast cancer (*n* = 36), and evaluated the mitotic status of each cell to assess the prognostic value of enumerated CTCs and their mitotic indices. These data suggest that applying histology-based mitotic indices to CTCs enhances patient stratification, and may provide an improvement in their prognostic significance.

## Methods

### Blood sample collection

In total, whole peripheral blood samples were drawn prospectively from 36 women who were actively undergoing treatment for previously confirmed stage III or stage IV breast cancer, at either the Fox Chase Cancer Center (FCCC) or University of Maryland, Baltimore (UMB) between 2011 and 2013. The study group characteristics can be found in Additional file 1: Table S1. Anonymized peripheral blood samples were supplied through a collaboration agreement with FCCC and UMB, with written informed consent and according to the local Institutional Review Board (IRB) approval at Fox Chase Cancer Center or University of Maryland Baltimore. In addition, healthy female volunteers (*n* = 16; median age 52 years), donated blood samples with written informed consent and approval from Western Institutional Review Board. All anonymized blood samples were drawn into CellSave preservative tubes™ (approximately 9 mL; Janssen Diagnostics): 7.5 mL of blood was used to enumerate CTCs using CellSieve™ microfiltration at UMB, FCCC or Creatv Microtech. Results and patient identification from institutions were not shared or communicated until completion of study.

### CellSieve™ low-flow microfiltration procedure

**Fig. 1:**
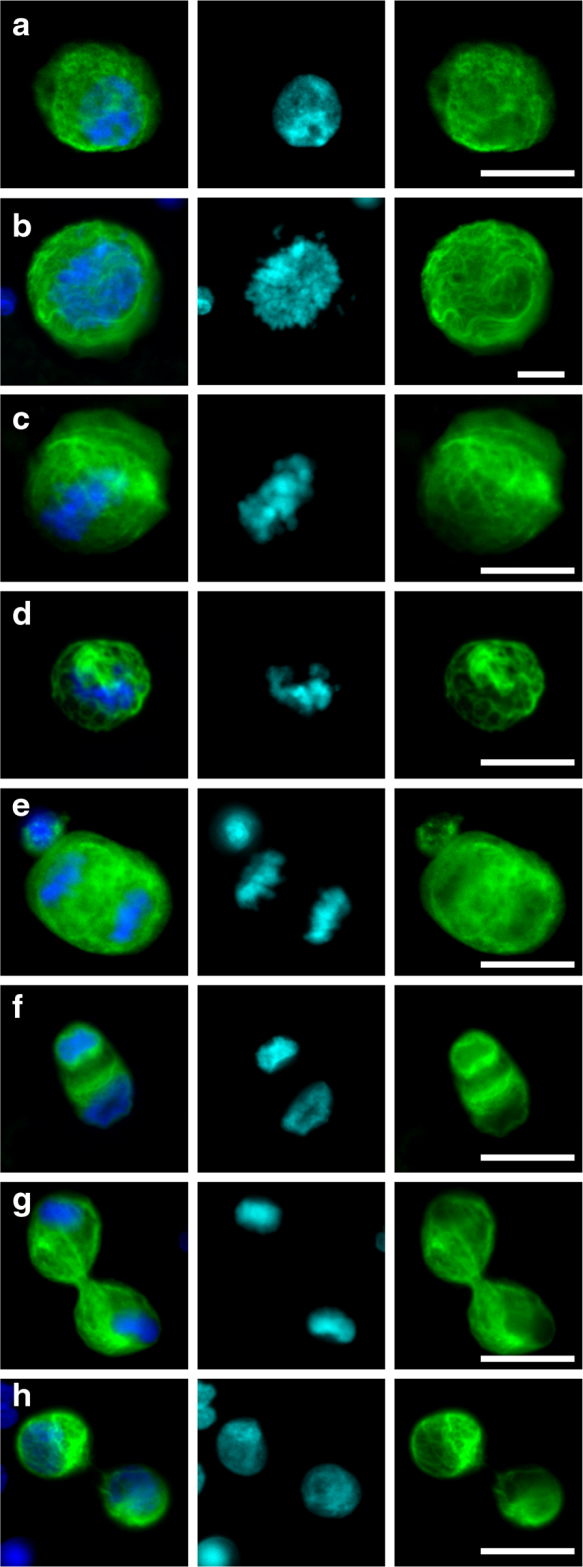
Common cytology of circulating tumor cells (CTCs) in mitosis, isolated from patients with breast cancer. Representative images of **a** the “classical” CTC in interphase (the most commonly found morphology), **b** prophase CTC with condensing chromatin, **c** metaphase CTC with condensed chromatin lining up along the axis, **d** metaphase/anaphase where two chromatins are separating along the cell plate, **e** anaphase where two chromosome sets are moving to cell poles, **f** telophase showing two cell envelopes and a contractile ring at the center of the cell, **g** cytokinesis showing the contractile ring pinching the cell into two, and **h** late cytokinesis during which the nuclear envelopes are reformed, contractile ring almost complete and chromatin has expanded. *Blue* 4',6-diamidino-2-phenylindole-positive nuclei, *green* cytokeratin. *Scale bar* 15 μm

Samples were run at FCCC, UMB or Creatv Microtech with a CellSieve™ Microfiltration Assay using a low-pressure vacuum system [18]. CellSieve™ Microfiltration Assay isolates CTCs based on size exclusion >7 μm, then a trained cytologist identifies CTCs based on the morphological features and the phenotypic expression of EpCAM, Cytokeratins 8, 18, and 19, and 4',6-diamidino-2-phenylindole (DAPI) (Fig. 1 and Additional file 1: Figures S1 and S2 (Additional file 1 is available in the online version of this paper)). Briefly, a low-pressure vacuum with CellSieve™ microfilters on a filter holder assembly is placed onto a waste apparatus. Whole peripheral blood (7.5 mL) collected in CellSave preservative tubes™ is prefixed, drawn through the filter (approximately 3 minutes), washed with PBS, post-fixed, and permeabilized. The filter is stained with an antibody cocktail of FITC-anti-Cytokeratin 8, 18, and 19; Phycoerythrin (PE)-EpCAM; and Cy5-CD45 [3, 17, 18]. Filters are washed and slide mounted with Fluoromount-G/DAPI (Southern Biotech). Pathologically definable CTCs (PDCTCs) are morphologically identified using pre-established cytological features as previously described [3]. An Olympus BX54WI fluorescent microscope with Carl Zeiss AxioCam and Zen2011 Blue (Carl Zeiss) was used to image cells.

### Enumerating CTCs

We have previously defined the various CTC subtypes isolated from patients with breast cancer. For this study, only intact cells that have pathologically definable characteristics (PDCTCs), as previously described [3], were counted as CTCs in this study. This includes CTCs that are CD45-negative, have a strong filamentous cytokeratin signal and have DAPI-positive nuclei with malignant pathological criteria. PDCTCs were identified and imaged by a trained cytologist and confirmed by a pathologist [3]. Apoptotic CTCs, CTCs undergoing epithelial to mesenchymal transition (i.e., absence of cytokeratin), and CTCs that could not be cytologically identified as malignant were not included in this study [3, 24].

### Grading mitotic proliferation

Mitosis was identified by a trained cytologist and confirmed by a pathologist. The stages of active mitosis, including prophase, prometaphase, metaphase, anaphase, telophase and cytokinesis, are all well-described using both nuclear and cytokeratin structures [5–8]. CTCs were only counted as mitotic if the cytologist could identify the cell was in a stage of M phase, otherwise the CTC was counted as non-mitotic (Additional file 1: Figures S1 and S2).

### Statistical methods

Kaplan-Meier estimates and Cox proportional hazard regression analyses were performed in Matlab R2013A using the enumerated CTC counts from all subtypes and the known patient populations. For survival analysis, the time to death was defined as the interval between when blood sample was obtained until death, or censored by last follow up visit. Estrogen receptor (ER), progesterone receptor (PR) and human epidermal growth factor receptor-2 (HER2) status were determined according to local guidelines. HER2 was considered positive at a value equal to or greater than 2+ (Additional file 1: Figure S3 and Additional file 1: Table S1). Cancer subtype, hormone status and stage were determined at the time blood was obtained. A power analysis (1-β = 0. 9, α = 0.05) determined that a sample size of 30 patients was sufficient to stratify patient cohorts based on previous CTC data analysis [3].

## Results

CTCs were found in 83 % of patient samples and in none of the healthy control samples, consistent with published studies (i.e., CellSearch® identifies CTCs in approximately 65–80 % of late-stage breast cancer) [1, 2, 4, 19, 20]. The majority of CTCs (approximately 91 %), identified by the differential staining of cytokeratin-positive, DAPI-positive and CD45-negative, had a malignant appearance, i.e., high cytoplasmic to nuclear ratios, high pleomorphism, and well-structured filamentous cytokeratin (Fig. 1, Additional file 1: Figures S1 and S2) [3, 4, 17–20].

**Fig. 2:**
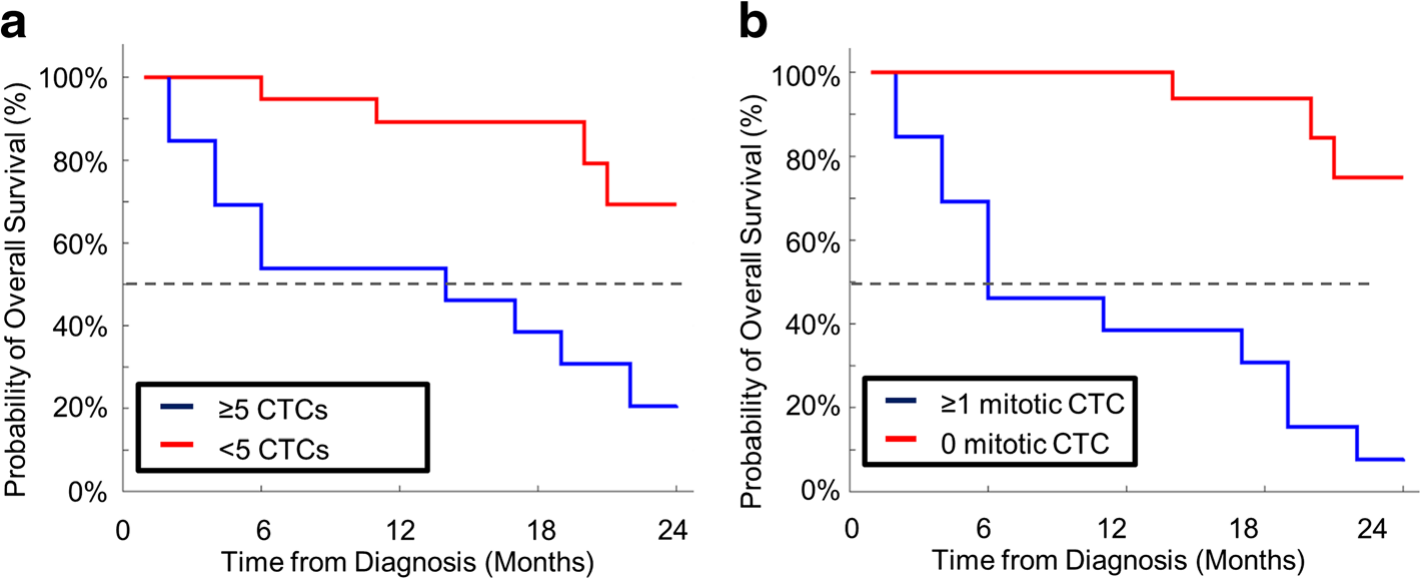
Kaplan-Meier estimates of probabilities of overall survival in the breast cancer patient populations, total circulating tumor cell (*CTC*) count versus mitotic CTC count (*n* = 36). **a** Kaplan-Meier estimates of probabilities of overall survival of patients with breast cancer with <5 CTCs per sample vs patients with ≥5 CTCs per sample. **b** Kaplan-Meier estimates of probabilities of overall survival of patients with breast cancer with 0 mitotic CTCs per sample vs patients with ≥1 mitotic CTCs per sample

We divided the patient cohort into subsets using the standard clinical cut off of ≥5 CTCs/sample to determine patient survival [1–4, 17–20]. Specifically, 23 of 36 patients (64 %) had <5 CTCs, with a median survival of >24 months, whereas 13 of 36 patients (36 %) had ≥5 CTCs, with a median survival of 10.0 months, hazard ratio 5.2 (Fig. 2a and Table 1). This hazard ratio was within the confidence interval of published ratios establishing ≥5 CTCs as the optimal cut off for evaluating patients (i.e., 26–49 % of patients with late-stage breast cancer have ≥5 CTCs per sample with reported median survival ranging from 10.1 to 15.0 months [1–4, 17–20]).

**Table 1:** Prediction table with the hazard ratios, confidence intervals and *p* values for the patient populations (*n* = 36)

Variable	Hazard ratio	95 % CI	*P* value
1 mitotic CTC vs 0 mitotic CTC	11.1	3.1-39.7	<0.001
≥5 CTC vs <5 CTC	5.2	1.6-16.5	0.005
1 mitotic CTC or ≥5 CTCs vs 0 mitotic CTCs and <5 CTCs	8.0	1.8-35.4	0.006
ER/PR-positive vs ER/PR-negative	1.3	0.5-3.7	0.174
HER2-positive vs HER2-negative	1.8	0.6-5.7	0.289
Hormone-positive vs hormone-negative (tn)	4.0	1.4-11.2	0.009

*CTC* circulating tumor cell, *ER* estrogen receptor, *PR* progesterone receptor, *HER2* human epidermal growth factor receptor-2, *Tn* triple negative

All CTCs were then sub-classified based on the cytological identification of M phase phenotypes [1–4, 17–20, 27]. We identified CTCs in all stages of mitosis from the patient cohort (Fig. 1b-h) [5, 6, 8, 27]. Specifically, 23 of 36 patients (64 %) had no mitotic CTCs, with a median survival >24 months, whereas 13 of 36 patients (36 %) had ≥1 mitotic events, with a median survival of 5.7 months, hazard ratio 11.1 (Fig. 2b, Table 1 and Additional file 1: Figure S4). Of note, mitotic events were detected in four patients with <5 CTCs and were not detected in four patients with ≥5 CTCs (Additional file 1: Figures S4 and S5). These data suggest that the additional visual characterization of mitosis in CTCs enhances the stratification of patients with breast cancer for prognostic correlation with survival, compared with CTC enumeration alone. In the re-stratified cohort, 92 % of patients with at least one mitotic CTC died within the 2-year period of observation vs 13 % of patients without mitotic CTCs, representing an 11-fold increase in patient risk (Table 1 and Fig. 2).

Of the 155 mitotic events identified, prophase was the most commonly observed (78 % of mitotic CTCs, or 121 cells), followed by telophase/cytokinesis (15 %, or 23 cells). Metaphase and anaphase were rarely observed, with only 4 in anaphase and 6 in metaphase, 2.6 % and 3.9 %, respectively. Interestingly, the frequency of mitotic events was more common than expected, at 9.3 % of all CTCs.

## Discussion

For many years, CTC research has attempted to differentiate clinically relevant CTCs from CTCs playing no role in metastatic spread, by analyzing mutation rates, proteomes, epithelial-to-mesenchymal transition, etc. [2, 17, 19, 20, 28], to improve their clinical utility. While it has been established that enumerating CTCs using the threshold of ≥5 CTC per sample is prognostically valuable, translating this information into direct treatment for improved patient survival has been difficult [1, 2, 12, 28]. Recently, protein level and genomic phenotyping of single CTCs has shown that they are a heterogeneous population of multiple complicated phenotypes [22, 28–30] and subtyping CTCs by this biomarker heterogeneity is an ongoing area of study. However, the complex heterogeneity, low numbers of CTC per sample, and the fact that 20–35 % of patients with late-stage cancer have no measurable CTCs, are all confounding factors inhibiting clinical utility [1, 2, 4, 12, 19]. In contrast to profiling CTCs with additional proteomic biomarkers, we analyzed cells using a more basic scientific approach, by identifying the cancer phenotype known to correlate with highly aggressive malignancies, i.e., mitosis, and known to provide predictive information about therapeutic response [5–9]. Using a sample size sufficient to properly stratify this patient cohort, our results determined that while CTC number is in fact a prognostic indicator of patient survival, by simply subtyping the same population based on CTCs in cytological mitosis, the hazard increased dramatically to 11.1. By identifying this highly aggressive CTC phenotype, we may now attempt to better understand these CTCs using more sophisticated molecular and proteomic targeting techniques, profiling these cells for mutations, stem cell properties, etc.

Using cytological assessment of CTCs, combined with the calculated patient survival, these observations imply that there are quantifiable populations of intact CTCs in mitosis found outside the tumor area, which could be the sought after clinically relevant CTC populations. While it cannot be determined whether these CTCs are actively dividing in the circulation or that dividing CTCs are simply breaking off the tumor into the circulatory system, the finding of these cells in late-stage breast cancer is intriguing. Considering that mitotic cells are less stable and prone to structural collapse, the stress of circulation should intuitively lessen the frequency of mitotic CTCs and destroy the cells before isolation, which did not occur [5–8]. Biologically, these events hint at aggressive cellular subtypes involved in the metastatic cascade, and our observations imply that there are quantifiable populations of CTCs in the mitotic phase found outside the tumor area.

Despite being a small cohort, the patient population represented patients with a heterogeneous group of breast cancers with a diverse hormone status (Table 1, Fig. 2 and Additional file 1: Table S1) and distinct cohort separation, indicating the possible applications to breast cancer in general. While these observations must now be expanded to include a larger and more diverse population of cancer patients, the sample size used in this study is more than sufficient to justify further testing for this CTC subtype in patients with late-stage breast cancer (Fig. 2 and Table 1). The presence of mitotic CTCs, and the association with increased risk, indicates the existence of a statistically significant cohort with an aggressive cancer subtype, dictating the need to further research this biological event and expand this study to a larger group of women.

## Conclusion

We suggest that tracking these mitotic cancer cells transiting the circulatory system provides a simple noninvasive method to gather clinical information on highly aggressive tumor cells and possibly aid the planning of patient treatment as tumor progression evolves. While tumor “omics” profiling promises a future of personalized treatment, the spread of disease (i.e., stage) followed by the aggressiveness of disease (i.e., grade) currently remain the first and second most important factors in a patient’s survival and treatment. Here, we suggest that incorporating mitotic indices into CTC assessment might better stratify patients into prognostic groups, better inform a physician of tumor evolution, and identify the more aggressive cancer targets using a blood-based biopsy.

## Additional file

Additional file 1: Figure S1. Common recognizable Cytologies of CTCs in Mitosis isolated from breast cancer patients with all the “standard” CTC stains from Fig. 1. **Figure S2.** Common recognizable Cytologies of CTCs in Mitosis isolated from breast cancer patients with all the “standard” CTC stains from Fig. 1. **Figure S3.** Kaplan-Meier estimates of probabilities of Overall Survival of the patient subpopulations based on receptor status from Fig. 2a (n=33). **Figure S4.** Box plot of total number of CTCs in each patient versus mitotic CTCs for each patient. **Figure S5.** CTC counts and Mitotic CTC counts for each patient sample in relation to time of filtration after blood draw. **Table S1.** Patient subpopulations classified by stage, receptor status and treatment. (PDF 748 kb)

## Abbreviations

CI: confidence interval
CTC: circulating tumor cell
DAPI: 4',6-diamidino-2-phenylindole
ER: estrogen receptor
FCCC: Fox Chase Cancer Center
HER2: human epidermal growth factor receptor-2
IRB: Institutional Review Board
MI: mitotic index
PBS: phosphate-buffered saline
PCNA: proliferating cell nuclear antigen
PDCTC: pathologically definable circulating tumor cell
PI: proliferation index
™: trademark
PR: progesterone receptor
UMB: University of Maryland Baltimore
α: alpha
β: beta
