# Supplementary material for: Mitosis in circulating tumor cells stratifies highly aggressive breast carcinomas
Source: Breast Cancer Res. 2016 May 4;18:44. doi: 10.1186/s13058-016-0706-4 (PMC4855427; doi:10.1186/s13058-016-0706-4)
Supplement: Additional file 1: Figure S1. — Common recognizable Cytologies of CTCs in Mitosis isolated from breast cancer patients with all the “standard” CTC stains from Fig. 1. Figure S2. Common recognizable Cytologies of CTCs in Mitosis isolated from breast cancer patients with all the “standard” CTC stains from Fig. 1. Figure S3. Kaplan-Meier estimates of probabilities of Overall Survival of the patient subpopulations based on receptor status from Fig. 2a (n=33). Figure S4. Box plot of total number of CTCs in each patient versus mitotic CTCs for each patient. Figure S5. CTC counts and Mitotic CTC counts for each patient sample in relation to time of filtration after blood draw. Table S1. Patient subpopulations classified by stage, receptor status and treatment. (PDF 748 kb) [file 13058_2016_706_MOESM1_ESM.pdf]

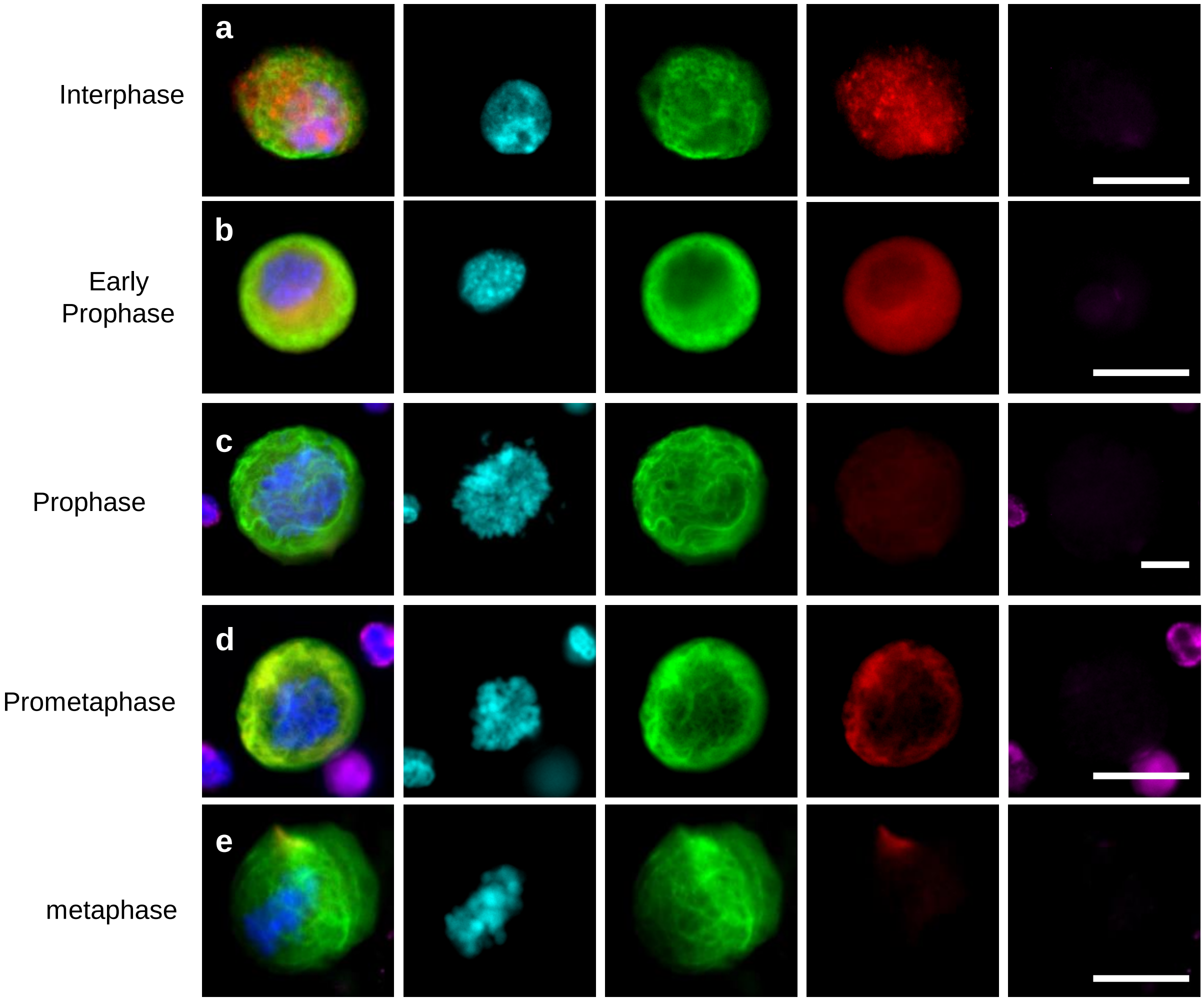

Supplementary Figure 1. Common recognizable Cytologies of CTCs in Mitosis isolated from breast cancer patients with all the "standard" CTC stains from Figure 1. (a) "Classical" EpCAM positive CTC in Interphase, i.e. not in mitosis (b) CTC in Early Prophase with chromatin condensing seen as small dots in the nucleus. (c) CTC in Prophase with condensed chromatin and highly active mitochondria seen outside the nucleus as DAPI dots (d) CTC in Late Prophase chromatin can be seen condensing to the center of the CTC (e) CTC in Metaphase with condensed chromatin lining up along the cell's axis. Scale bar=15 μm

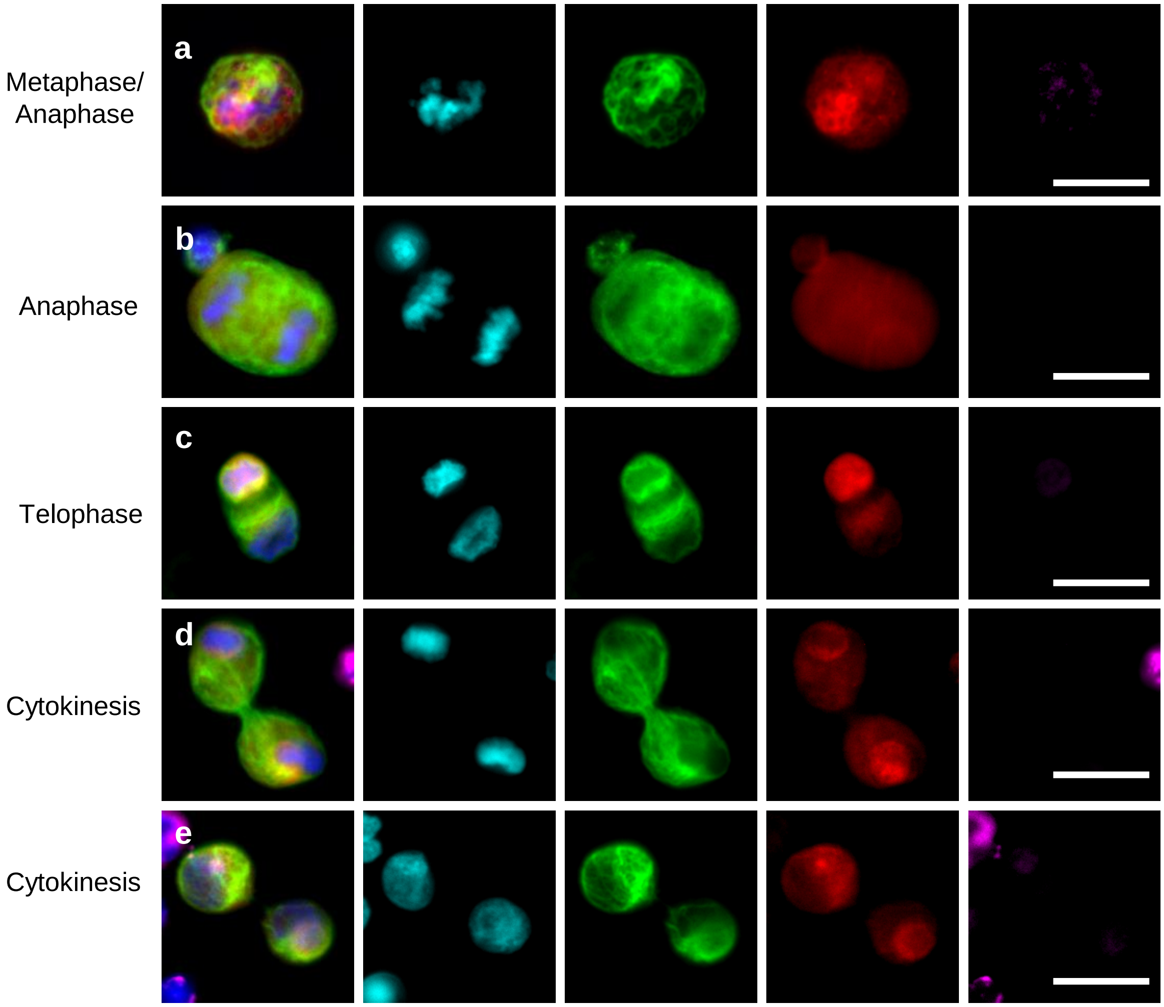

Supplementary Figure 2. Common recognizable Cytologies of CTCs in Mitosis isolated from breast cancer patients with all the "standard" CTC stains from Figure 1. (a) CTC in Metaphase/Anaphase transition, the two chromatins can be seen beginning to separate along the cell plate a cell plate can be seen along the cell's axis. (b) CTC in Anaphase with the two chromosome sets seen moving to polar ends of the cell. (c) CTC in telophase is seen with two distinct cell envelopes and a contractile ring in the center of the cell (d) Cell in Late telophase/Cytokinesis as a contractile ring is pinching the cell into two cells but the chromatin remains condensed. (e) End of Cytokinesis as nuclear envelopes are reformed, the contractile ring almost complete and the chromatin has expanded. Scale bar=15 µm

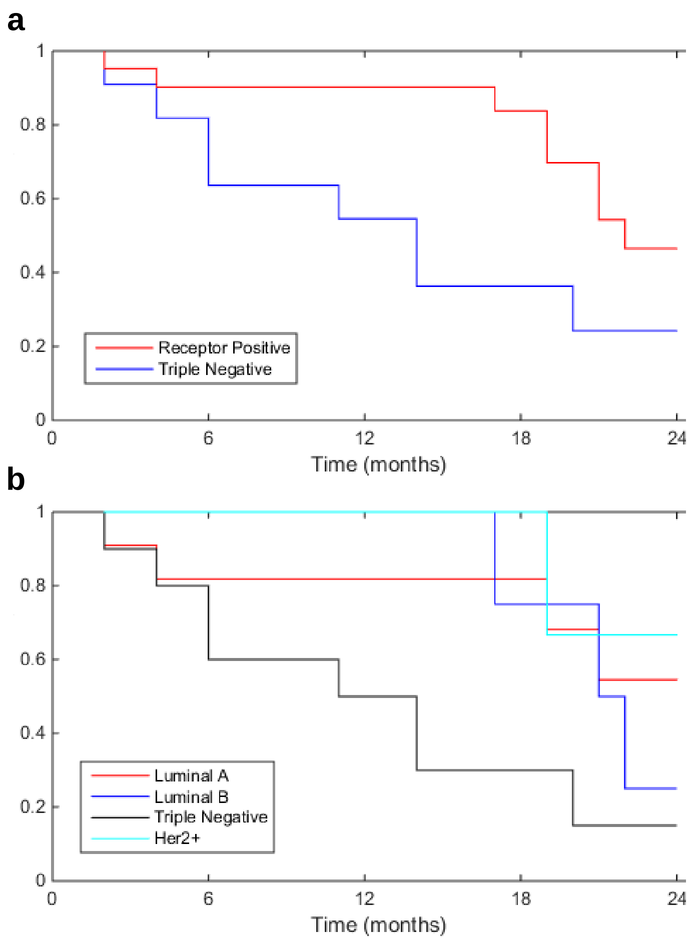

Hazard: 4.0
(CI95% 1.4-11.2)
P=0.01

Hazard:
*Lum. B: 1.5 (CI95% 0.-6.8)
p=0.59
*Tri Neg: 3.1 (CI95% 0.9-10.5)
p=0.08
*HER2+: 0.7 (CI95% 0.3-2.3)
p=.061

Supplementary Figure 3. Kaplan-Meier estimates of probabilities of Overall Survival of the patient subpopulations based on receptor status from Figure 2a (n=33). Three of the 36 patients did not have know receptor status. *All hazard ratios were calculated based on the Luminal A patient cohort.

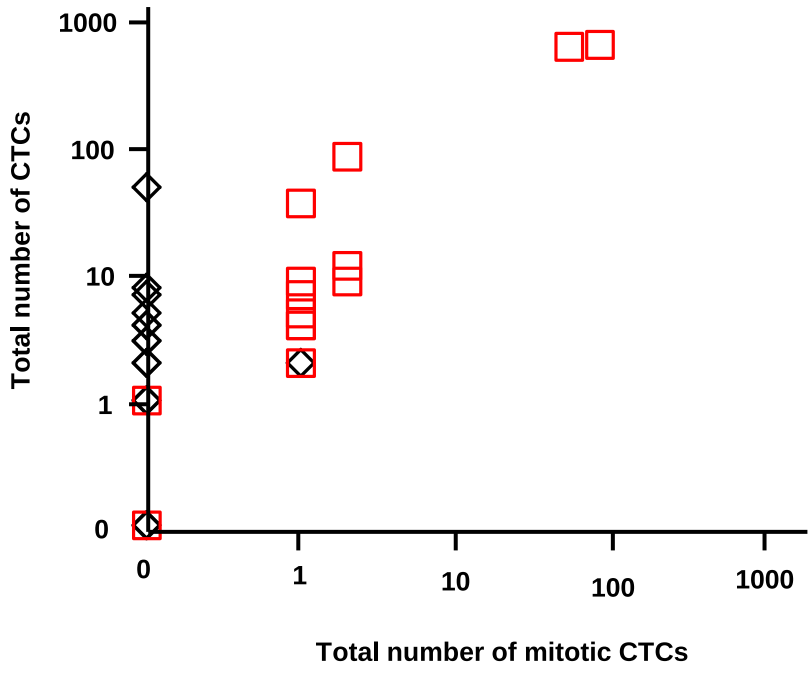

Supplementary Figure 4. Box plot of total number of CTCs in each patient versus mitotic CTCs for each patient. Red squares=patients expired within 2 years, black diamonds=alive patients within 2 years

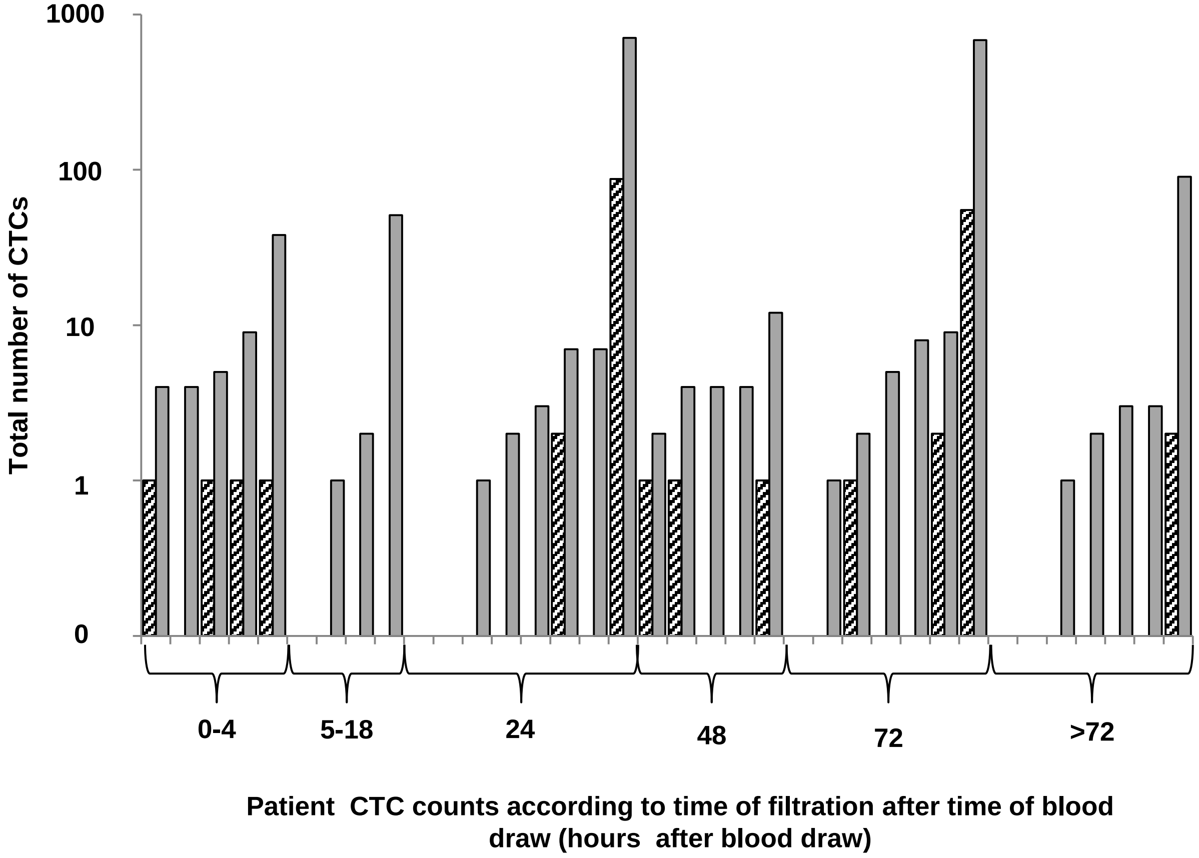

Supplementary Figure 5. CTC counts and Mitotic CTC counts for each patient sample in relation to time of filtration after blood draw. Solid bars=Total CTC count, Striped bars=mitotic CTC count.

		Number of patients
Stage	1	-
	2	-
	3	9
	4	27
ER/PR*	Positive	16
	Negative	17
HER2*	Positive	9
	Negative	24
ER/PR/HER2*	Positive	23
	Negative	10
Treatment	Baseline	5
	1st Line	9
	2nd Line, or subsequent†	22
Pathological Grade	1	1
	2	6
	3	19
	‡unknown	10
Histology	Ductal	17
	Lobular	3
	‡other	16

Supplementary Table 1. Patient subpopulations classified by stage, receptor status and treatment.

*3 of 36 patients had unknown receptor type

†8 patients were not currently on therapy, but starting 2nd line treatment.

‡ Tissue unavailable for histology assessment, and/or patients with unspecified metastatic breast cancer, and/or patients with cancers other than IDC or ILC.
